# PAPILA: Dataset with fundus images and clinical data of both eyes of the same patient for glaucoma assessment

**DOI:** 10.1038/s41597-022-01388-1

**Published:** 2022-06-09

**Authors:** Oleksandr Kovalyk, Juan Morales-Sánchez, Rafael Verdú-Monedero, Inmaculada Sellés-Navarro, Ana Palazón-Cabanes, José-Luis Sancho-Gómez

**Affiliations:** 1 Universidad Politécnica de Cartagena, 30202 Cartagena, Spain; 2 Hospital General Universitario Reina Sofía, 30003 Murcia, Spain

**Keywords:** Databases, Medical imaging, Optic nerve diseases

## Abstract

Glaucoma is one of the ophthalmological diseases that frequently causes loss of vision in today’s society. Previous studies assess which anatomical parameters of the optic nerve can be predictive of glaucomatous damage, but to date there is no test that by itself has sufficient sensitivity and specificity to diagnose this disease. This work provides a public dataset with medical data and fundus images of both eyes of the same patient. Segmentations of the cup and optic disc, as well as the labeling of the patients based on the evaluation of clinical data are also provided. The dataset has been tested with a neural network to classify healthy and glaucoma patients. Specifically, the ResNet-50 has been used as the basis to classify patients using information from each eye independently as well as using the joint information from both eyes of each patient. Results provide the baseline metrics, with the aim of promoting research in the early detection of glaucoma based on the joint analysis of both eyes of the same patient.

## Abstract (summary)

**Table:** 

Measurement(s)	Retinal fundus image • Refractive defect • Intraocular pressure • Central corneal thickness • Axial length • Visual field
Technology Type(s)	Non-mydriatic retinal camera • Autorefractometer/keratometer • Non-contact tonometer • Specular Microscope Pachymeter • Optical biometry • Field Analyzer/campimeter

## Background & Summary

Glaucoma is a progressive disease of the Optic Nerve Head (ONH) caused by high Intra-Ocular Pressure (IOP) due to poor drainage of ocular fluid^1^. Clinically, it is a silent ocular disease which produces a progressive and irreversible deterioration of the visual field that progresses to a total loss of vision. Glaucoma is currently the second leading cause of blindness in the world, affecting one in every two hundred people under fifty years of age and one in ten over eighty years^2^. In 2040, 111.8 million people aged 40–80 years are estimated to suffer from glaucoma^3^.

Glaucoma is initially an asymptomatic (but preventable) disease, for this reason it is generally detected in very advanced stages when the loss of the visual field is very manifest and irreversible. At the moment, there is no cure for established glaucomatous damage, and therefore early detection and prevention is the only way to avoid the progression to a total blindness. The development of tools for the detection of glaucoma in its early stages is essential to preserve eye health as well as minimize health costs.

The indicators for the diagnosis of this disease appear years before irreversible lesions in the visual field. The main procedures for the diagnosis of glaucoma are tonometry to assess the intraocular pressure, campimetry to assess the visual field, and retinal fundus images to assess characteristics of the optic nerve head^2^. Among them, Retinal Fundus Images (RFI) is a biomedical imaging modality^4,5^ which is cost-effective, non-expensive and non-invasive, suitable for screening ophtalmic diseases such as glaucoma. RFIs provides morphological parameters to identify the onset of glaucoma and follow-up on its progression.

The parameters of the ONH which can be analyzed from fundus images are based on the size and shape of the Optic Disc (OD), Optic Cup (OC), and neuroretinal rim. Specifically, the relation between the diameter of the OC and the diameter of the OD provides the Cup-to-Disc Ratio (CDR)^6^, the relation between the minimum rim-width and the diameter of the OD provides the Rim-to-Disc Ratio (RDR)^7^, and the neuroretinal rim provides the width pattern Inferior ≥ Superior ≥ Nasal ≥ Temporal, known as the ISNT rule^8,9^. As glaucoma progresses, the cup becomes larger, usually due to the raising of the intraocular pressure, resulting in an increase of the CDR value, a decrease of the RDR value over time and the violation of the ISNT rule. The measurement of these parameters can be done manually by experts or by automatic methods based on image processing^10,11^ and/or machine learning^12,13^.

In the last decade, research efforts have been directed mainly towards methods based on deep learning^14^, which have proven to be very effective in image classification and segmentation tasks, achieving very promising results in the field of ophthalmology^13^. For current reviews of existing methods to aid in the diagnosis of glaucoma, the reader is invited to consult the works of, e.g., Thakur^15^ or Almazroa^16^. These methods generally need a large and properly-labeled dataset to train their machine learning models, and the success of the model working in operation mode depends directly on the quality and quantity of the training dataset^17^. In general, building a dataset is a time-consuming task, which becomes more tedious when it handles medical data from several sources. As detailed below and in Table 1, particularly in the diagnosis of glaucoma, there are some datasets only with RFI (not always properly diagnosed), and others dataset which also provide the segmentation of the optic disc, optic cup or both.

**Table 1:** Comparison of the PAPILA dataset with other publicly available datasets.

Dataset	Number of images			Ground truth labels			Diagnosis from	Both eyes of the same patient
	Total	Healthy	Glaucoma (or suspect)	Glaucoma classification	Optic disc contour	Optic cup countour		
RIGA^28,29^	750	—	—	✗	✓	✓	—	✗
ORIGA^30^	650	482	168	✓	✓	✓	Not specified	✗
RIMONE^31,34^	485	313	172	✓	✓	✓	Clinical	✗
Drishti-GS^35,36^	101	70	31	✓	✓	✓	Image	✗
ACRIMA^37,38^	705	309	396	✓	✗	✗	Image	✗
G1020^39,40^	1020	724	296	✓	✓	✓	Clinical	✗
REFUGE^13,41^	1200	1080	120	✓	✓	✓	Clinical	✗
PAPILA^44^	488	333	155	✓	✓	✓	Clinical	✓

Note that in this summary the *suspect* class and *glaucoma* class in PAPILA dataset have been merged, as in the case of RIMONE dataset, for comparative purposes.

Neither the datasets described below nor any other that had been located by the authors contains clinical data as well as retinal fundus images of both eyes of the same patient. For this reason, the proposed PAPILA dataset intends to be useful for developing algorithms which learn and discover other supplemental manifestations using the joint information of both eyes for the early diagnosis of glaucoma, that could be too difficult to notice considering an isolated eye.

## Methods

This section details existing databases and describes the proposed dataset explaining the criteria followed in the design and the reasons for including or not including patients in it. The description of every clinical data gathered for each patient is also detailed.

### Existing databases

There are widely used and referenced datasets with RFI such as, e.g., DRIVE^18,19^, which contains 40 fundus images and deals with diabetic retinopathy providing the segmentation of blood vessels; DiaRetDb1^20,21^, with 89 color fundus images with their segmentation and annotated information for different diabetic retinopathies; the STARE project^22,23^ with 400 labeled images, as well as the segmentation of the optic nerve in 80 images; DRIONS^24,25^ with no-labeled 110 images and two segmentations of the optic disc for each one. Focused specially on glaucoma, some of the most known datasets are the following:

- The Messidor project^26,27^, whose main purpose is to compare and evaluate segmentation algorithms developed for the detection of lesions in color retinal images. This dataset contains 1200 fundus images with its corresponding medical diagnosis.
- RIGA^28,29^ is a dataset for glaucoma analysis with 750 retinal fundus images. The dataset provides the optic cup and optic disc boundaries for each image but the glaucoma diagnosis is not given.
- ORIGA^30^ is composed of 482 images of healthy patients and 168 images from patients with glaucoma, together with the segmentation of the disc and cup. This dataset was public and downloadable in 2010 but at this moment it seems not to be longer publicly available.
- The public dataset RIMONE^31^ was firstly released in 2011^32^. Four years later, in 2015, 159 stereo fundus images with two ground truth segmentations of disc and cup were provided to assess the CDR^33^. These images corresponded to healthy and glaucoma patients. Recently, in 2020, the dataset has been revisited and optimized for a deep-learning context^34^. The updated dataset contains 313 retinographies from normal subjects and 172 retinographies from patients with glaucoma.
- Drishti-GS^35,36^ is a publicly available dataset for glaucoma assesment with optic disc and cup segmentations. It consists of 101 monocular fundus images (70 images of glaucoma and 31 normal images), split in training and test sets, with four expert segmentations of the disc and cup for the training set.
- ACRIMA^37,38^ contains 705 labelled public fundus images (396 glaucomatous images and 309 normal images). The annotations were made by two glaucoma experts and no other clinical information was taken into account while providing labels for the images.
- G1020^39,40^ is a large retinal fundus image dataset with 1020 publicly available fundus images (724 healthy and 296 glaucoma) for glaucoma diagnosis. Labeling of the images, as well as optic disc and optic cup segmentation is provided.
- The recent REFUGE dataset^13,41^ contains 1200 fundus images with ground truth segmentations of the optic disc and optic cup, and clinical glaucoma labels (120 images from patients with glaucoma and 1080 images from healthy patients).

### Selection of patients

The PAPILA dataset was collected at the *Department of Ophthalmology* of the *Hospital General Universitario Reina Sofía*, HGURS, (Murcia, Spain) between years 2018 and 2020. This study was carried out in accordance with the tenets of the Declaration of Helsinki and with the approval of the hospital’s Ethics Committee. After signing an informed consent, the patients were divided into two groups: In *Group 1*, patients diagnosed with simple chronic glaucoma recruited in the *Glaucoma Area* of the HGURS; and in *Group 2*, patients from primary care who, after a ophthalmological examination, did not show any ocular pathology that could influence the morphology of the optic nerve.

The following medical data of both eyes was collected from all patients: refractive error, intraocular pressure, central corneal thickness (used to adjust IOP according to pachymetry), axial length and a color fundus image. In addition, when patients of *Group 2* had IOP greater than 22 mmHg, the Visual Field (VF) was also retrieved using the 30-2 program^42^. It is considered that a visual field has lesions suggestive of glaucoma when, complying with the confidence indices, at least three points in the same hemifield are observed with values 5% below of normal, excluding the pericecal and peripheral rows. In these cases, the VF was repeated, and glaucomatous damage was diagnosed if the defects were consistent. Table 2 gathers this medical data and details the models of each acquisition device.

**Table 2:** Summary of acquisition devices employed in the PAPILA dataset to collect medical data.

Medical data	Acquisition device	Model
Retinal fundus image	Non-mydriatic retinal camera	Topcon TRC-NW400
Refractive defect	Autorefractometer/keratometer	Nidek ARK-710 A
Intraocular pressure	Non-contact tonometer	Nidek NT-2000
Central corneal thickness	Specular Microscope Pachymeter	Rodenstock REM 3000
Axial length	Optical biometry	Zeiss IOL Master 500
Visual field	Field Analyzer/campimeter	Zeiss Humphrey 750i

Regardless the group, patients with opacities in the transparent media (corneal alterations and advanced cataracts) that prevented obtaining an assessable fundus image were excluded from the study. The alteration in the confidence indices of the VF was another reason for excluding a patient if the alteration persisted in two consecutive tests.

According to the ophthalmological examination, patients in *Group 2* were further classified into: *Group 2.a*, with individuals without glaucoma-related ocular pathology and whose IOP was less or equal than to 22 mmHg; *Group 2.b* with ocular hypertensive individuals, whose IOP ranged between 22–28 mm Hg without visual field affectation. No patients with IOP greater than 22 mmHg and lesions suggestive of glaucomatous neuropathy were detected.

The sample size of patients belonging *Group 2* was obtained considering the population of *Area VII* of the city of Murcia and the prevalence of simple chronic glaucoma (3,5% in the population older than 40 years), applying a confidence level of 95% and a 3% margin of error.

### Composition of the dataset

**Fig. 1:**
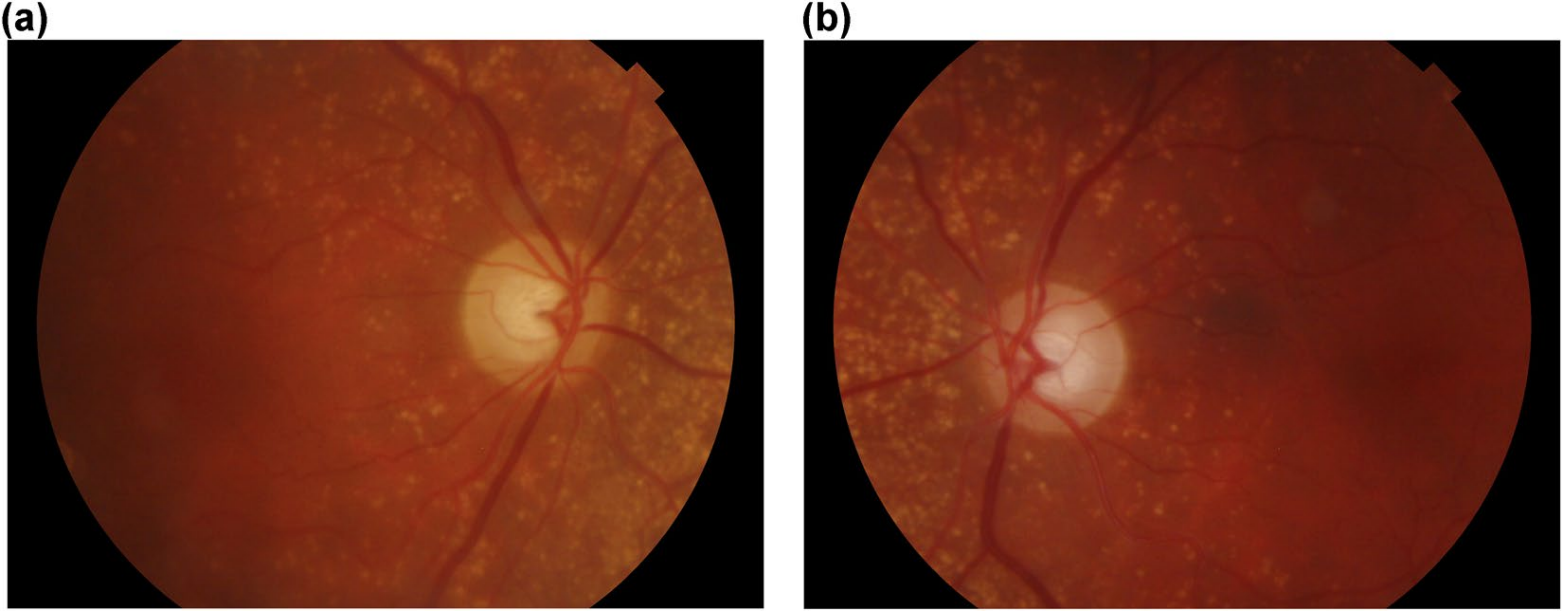
Retinal fundus images of both eyes of the same patient. Consent was acquired from the individual to depict their image. (**a**) Right eye of Patient #47 (*RET047OD.jpg*), (**b**) Left eye of Patient #47 (*RET047OS.jpg*).
- Knowledge transferred by ophthalmologists:

The proposed PAPILA dataset contains records of 244 patients. Each record provides structured information about clinical data, optic disc and optic cup segmentations of both eyes of the same patient. Labeling with the diagnosis is also provided considering clinical data. The records were properly anonymized and an unique identifier was assigned to each record. More specifically, each record contains:

- Age and gender of the patient.
- RFI of both the left and right eye, centered at the papilla with an aperture of 30°, in JPEG format, with 8 bits per color channel (see Fig. 1). These images have been acquired by ophthalmologist or technicians in the HGURS (Murcia, Spain), using a Topcon TRC-NW400 non-mydriatic retinal camera with a resolution of 2576 × 1934 pixels.

- Trustworthy labeling of the patient. Three cases are considered: glaucomatous, non-glaucomatous and suspect. The diagnostic labels were assigned based on the comprehensive evaluation of the clinical data of the subject (sometimes with a retrospective analysis of clinical records). Table 4 shows the distribution of the type of patients detailing gender and age ranges.

**Table 3:** Correction factor to add to IOP for a given corneal thickness^43^.

Thickness (*μ*m)	475	485	495	505	515	525	535	545	555	565	575	585	595	605	615
Factor (mmHg)	+5	+4	+4	+3	+2	+1	+1	0	−1	−1	−2	−3	−4	−4	−5

**Table 4:** Distribution of the type of patients in the PAPILA dataset according to gender and age ranges.

Age range	Gender	Healthy		Glaucoma		Suspect		Total
		Right eye	Left eye	Right eye	Left eye	Right eye	Left eye	
14–24	Male	0	0	0	0	1	1	**2**
	Female	0	0	0	0	1	1	**2**
25–35	Male	4	4	0	0	0	0	**8**
	Female	4	4	0	0	0	0	**8**
36–46	Male	11	11	0	0	1	1	**24**
	Female	11	11	0	0	0	0	**22**
47–57	Male	11	11	2	2	10	10	**46**
	Female	32	31	2	3	5	5	**78**
58–68	Male	21	20	7	8	3	3	**62**
	Female	36	35	5	6	5	5	**92**
69–79	Male	9	7	7	9	4	4	**40**
	Female	26	24	12	14	2	2	**80**
80–90	Male	1	1	0	0	1	1	**4**
	Female	4	4	5	5	1	1	**20**
**Total**		**170**	**163**	**40**	**47**	**34**	**34**	**488**

The count has been made eye-wise since the diagnosis is made for each eye separately.
- Segmentations of the OD and OC in RFI of both eyes. Manual annotations provided by two expert ophtalmologists with two (Expert #1) and twenty seven (Expert #2) years of experience, from the *Department of Ophthalmology* at HGURS. The annotation procedure consisted in manually placing points (pixel-wise) linked with lines to delineate the contours of the OD ad OC, separately, with an own developed tool with capabilities for image review, zoom and contour editing.

- Clinical data and medical test results:

- Refractive error. Vision problem that happens when the shape of the eye does not bend light correctly and keeps light from focusing correctly on the retina, resulting in a blurred image. The main types of refractive errors are myopia (nearsightedness), hyperopia (farsightedness), presbyopia (loss of near vision with age), and astigmatism. A person with myopia would have a negative refractive error, a person with emmetropia would have zero refractive error and a person with hyperopia would have a positive refractive error. In the case of astigmatism associated with the previous defects, the refractive error is expressed with 3 values: sphere, cylinder and axis.
- Crystalline lens. This item informs if the eye has the crystalline lens (phakic) or if it has been surgically removed (pseudophakic).
- IOP of both eyes. Normal values for healthy patients range from 10 mmHg to 21 mmHg. Values of IOP are obtained using non-contact tonometer Nidek NT-2000 using the Pneumatic or Perkins method.
- Corneal thickness. This measurement is obtained by pachymetry with the specular microscope pachymeter Rodenstock REM 3000. The mean value in healthy patients is 540 *μ*m. This characteristic is relevant in patients with glaucoma since it perturbs IOP measurements. Depending on the corneal thickness, a correction factor (see Table 3) must be added to or subtracted from the IOP value^43^.
- Axial length. This is the distance between the anterior vertex (central area of the cornea) and the posterior pole of the eye (central area of the retina). The axial length of the eye is approximately 24 mm in adulthood. It is typically longer than 24 mm in myopes and shorter than 24 mm in hyperopes. This measurement has been obtained by optical biometry with the Zeiss IOL Master 500.
- Mean defect (MD) of both eyes. This parameter, equivalent to the Visual Field Index (VFI), has been measured with the Humphrey field analyzer Zeiss Humphrey 750i following the 30-2 strategy^42^ (assessing a grid of 76 points over the central 30° of the visual field). This measurement gives an overall value of the total amount of visual field loss compared to the normal sensitivity expected for the population group with the age of the patient. Normal values typically range from 0 dB to −2 dB. The MD value becomes more negative as the overall field worsens. The MD value can be helpful for monitoring visual field loss in moderate-stage glaucomatous patients (−6 dB to 12 dB). A patient with glaucoma and MD between −3 dB and −6 dB is classified as mild glaucoma, values between −6 and −12 dB is moderate glaucoma and above −12 dB is severe glaucoma (see Fig. 3). This test is retrieved only in glaucomatous patients from *Group 1*.

**Fig. 2:**
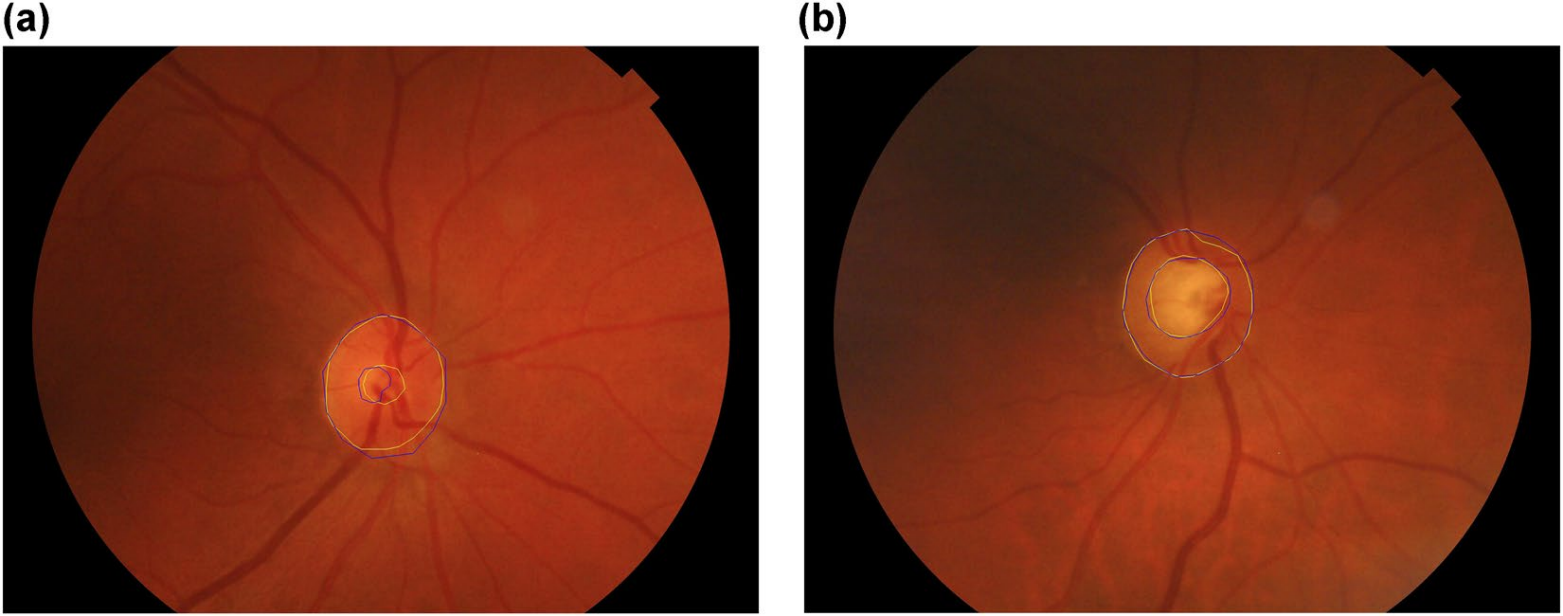
Manual segmentations of the optic disc and optic cup performed by two ophtalmologists. Consent was acquired from the individuals to depict their images. (**a**) Right eye of Patient #73, (**b**) Right eye of Patient #20.

**Fig. 3:**
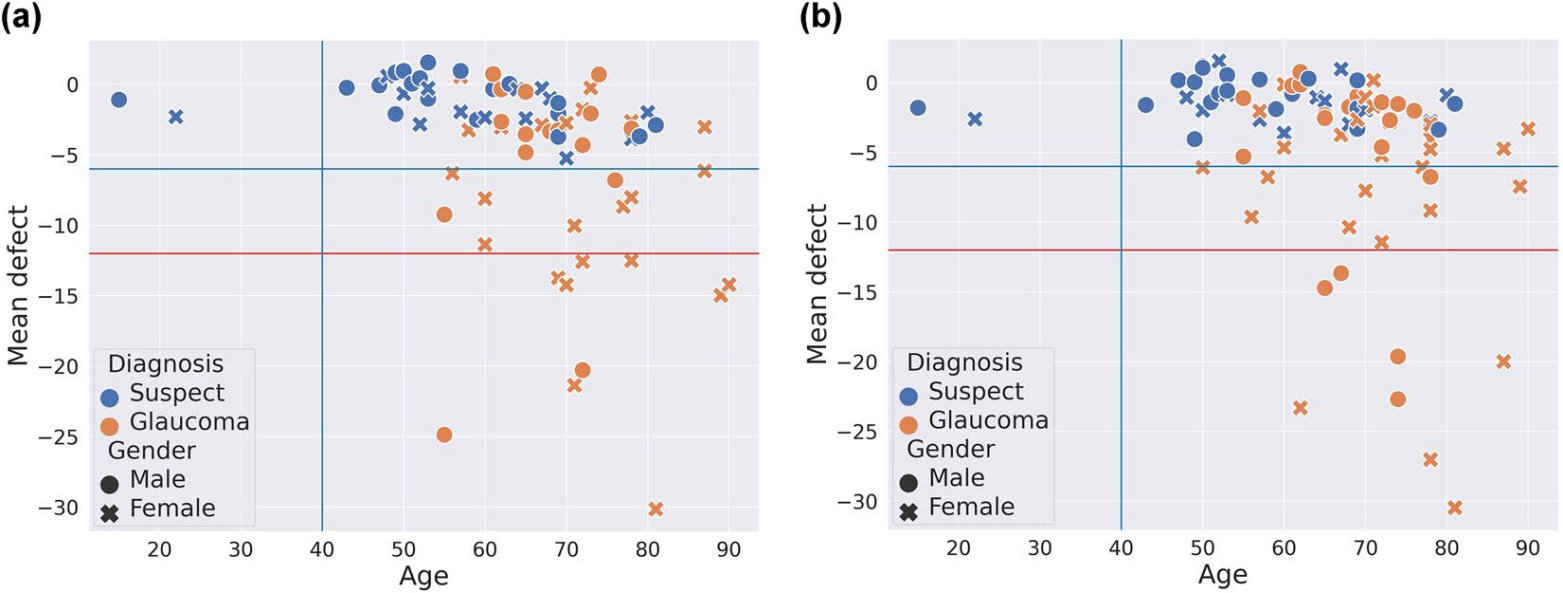
Distribution of the mean defect of the right eye and left eye versus the age of *suspicious* and *glaucoma* patients. Based on the mean defect value, three areas have been delimited with horizontal lines, corresponding to mild, moderate and advanced glaucoma. (**a**) Right eyes, (**b**) Left eyes.

## Data Records

The complete PAPILA dataset^44^ is available at the public figshare repository 10.6084/m9.figshare.14798004.v1. The dataset has a directory tree with the following structure:

The folder ClinicalData contains the clinical data and the diagnosis of 244 patients. The information of the patients is stored in spreadsheet format in two separate files, one for the right eye (OD) and one for the left eye (OS). The acronyms OD and OS refer, respectively, to the right eye and to the left eye in Latin, i.e., *Oculus Dexter* and *Oculus Sinister*. These files have the information organized in a table where each row corresponds to a patient and the columns contain the following fields: the unique patient identifier, the age of the patient, the gender of the patient (0 for male and 1 for female), the diagnosis (0 stands for healthy, 1 for glaucoma, and 2 for suspicious), the refractive error, phakic/pseudophakic (1 means that the crystalline lens has been removed and 0 means that the eye keeps the lens), the intraocular pressure, the pachymetry, the axial length, and the mean defect.

The next folder, ExpertsSegmentations, stores the segmentations of the optic disc and optic cup of the two eyes of each patient performed by the two ophthalmologists. There are 2 × 2 × 2 × 244 = 1952 files with the X and Y coordinates, in plain text, of the nodes of each contour. The names of the files have the following nomenclature: the prefix *RET*, three digits with the patient number, the string *OD* or *OS* indicating whether it is the right or left eye, the string *cup* or *disc* indicating which contour it is, and a final string *exp1* or *exp2* indicating the expert who performed the segmentation. For ease of understanding and illustrative purposes, this folder also contains the fundus images in JPEG format with the segmentation contours superimposed of both experts (see Fig. 2).

The folder FundusImages provides the 2576 × 1934 retinal fundus images of both eyes of all patients. There are 488 files in JPEG format corresponding to the right and left eye of 244 patients. The names of these files follow the same nomenclature as the files with the segmentations.

Finally, the folder HelpCode include some programs to help any researcher to handle the data provided.

## Technical Validation

### Validation of patient’s clinical data

Initially, the results of the ophthalmological tests were collected manually in the hospital from each of the devices described in Table 2, along with the fundus images. The test results were subsequently transcribed into a spreadsheet and carefully reviewed by ophthalmologists to verify that there were no outliers or inconsistencies in the data. Unfortunately, due to some impediments, a small set of records lacks certain irrecoverable information (Table 5), although it has the labeling with the diagnosis as well as the segmentations of the two experts. The authors have considered keeping these records because, although incomplete, they may be useful in classification methods with missing data^45,46^.

**Table 5:** Patients with missing data.

Patient ID	Missing Clinical Data
19, 20, 46, 57, 114, 116	Pachymetry
19, 48, 64, 79	Axial length
213, 218	Intraocular pressure
213	Refractive error
Healthy patients	Mean Defect

### Validation of expert segmentations

The segmentations of the optic disc and optic cup have been carried out by two ophthalmologists taking into account their deep knowledge and extensive experience, concretely, two and twenty seven years of experience in the specialty of Ophthalmology. However, due to the existence of a subjective component, the consistency of the segmentations has been measured using the Sorensen-Dice coefficient. For this purpose, a mask, i.e. a binary image with the same size as the RFI, has been generated from each contour with the pixels inside the contour set to one and the pixels that lie outside the contour set to zero. Then, the similarity between the contours made by the two experts has been computed as

1 $$D(A,B)=\frac{2| A\cap B| }{| A| +| B| },$$

where *A* and *B* are the corresponding binary masks and *D*(*A,B*) ranges from 0 (no similarity between the masks) to 1 (both masks are identical). To guarantee the quality of the segmentations, two minimum thresholds have been established in the measurements: 0.8 in the case of the optic disc segmentations, and 0.7 for the segmentations of the cup (except in the case of patients with very small optic cups).

To assess the interobserver and intraobserver variability in the segmentation of the optic disc and optic cup in retinal fundus images, the Sorensen-Dice similarity coefficient and the area-based cup-to-disc ratio (CDR)^47^ have been computed, performing student’s *t*-tests over the CDR measurements. The interobserver metrics consider both eyes of all the 244 patients in the database, i.e., 488 eyes, whereas the intraobserver metrics have been calculated with a random selection of 15 patients, i.e., 30 eyes, whose segmentations of the cup and optic disc have been done again by the experts two years after the first time.

Second and third columns of Table 6 gather the interobserver and intraobserver agreement between manual segmentations expressed as mean ± standard deviation of the Sorensen-Dice similarity coefficient. As can be seen, the agreement between the segmentations is greater with the optic discs than with the cups, both in the interobserver and intraobserver measurements. In general, the edges of the optic disc are clearly visible and its segmentation is easier than the segmentation of the optic cup, which requires expert knowledge and has a higher degree of subjectivity. Another result that can be deduced is that the intraobserver agreement is slightly higher than the interobserver, both in the disc and cup measurements.

**Table 6:** Interobserver and intraobserver agreement using the Sorensen-Dice similarity coefficient in expert segmentations of the cup and optic disc in retinal fundus images (second and third columns).

	Dice similarity coefficient		Difference of area-based CDR		
	Optic cup	Optic disc	mean ± std	*p*-value	CI
**Interobserver**	0.823 ± 0.089	0.935 ± 0.036	−0.002 ± 0.045	0.231	[−0.006, 0.001]
**Intraobserver**					
• Expert #1	0.827 ± 0.093	0.958 ± 0.037	0.002 ± 0.055	0.803	[−0.018, 0.023]
• Expert #2	0.834 ± 0.099	0.955 ± 0.033	0.008 ± 0.060	0.465	[−0.014, 0.030]

Statistical characterization of the interobserver and intraobserver variability using the difference in area-based CDR using the segmentations of the experts (forth to sixth columns).

In addition, the interobserver and intraobserver variability has been evaluated on the difference in the area-based CDR using the segmentations of the experts. The CDR is used in Ophthalmology to evaluate the evolution of glaucoma and compares the diameter of the cup with the diameter of the disc (the value of CDR ranges from 0 to 1). To determine if the means of two sets of measurements are significantly concordant from each other a student’s t-test have been performed. Forth to sixth columns of Table 6 show the results of these tests. The forth column of Table 6 contains the mean and standard deviation of the difference of the CDR for the interobserver and intraobserver cases, the fifth column provides the *p*-value associated to the corresponding matched measurements, and the last column shows the 95% confidence interval (CI) for the true mean of each test.

### Baseline results for supervised fundus image classification

To demonstrate the technical validity and give some insight into the statistical quality of the dataset, some direct experiments were performed. The proposed dataset can be used for machine learning purposes in a variety of tasks, with the diagnosis of patients being the most significant. The aim of these experiments is to provide baseline classification metrics that could be useful as a reference for future research. The primary diagnosis approach underlying in the experiments is based on deep Convolutional Neural Networks (CNNs), which have demonstrated to be effective methods for image classification. In the experiments a rectangular Region of Interest (ROI) that includes the optic disc was considered. These ROIs were cropped from original fundus images and resized to 200 × 200 × 3 before feeding the neural network. The segmentations of the optic disc made by *expert 1* (included in the proposed dataset) were used to determine the optic disc bounding box for every fundus image, and from them to extract a ROI of fixed and common size. This idea for ROI extraction is a common practice and diverse research^48,49^ can be found about fine or coarse optical disc segmentations in retinal images, which constitute satisfactory and automatic methods to achieve comparable ROI cropping results.

**Fig. 4:**
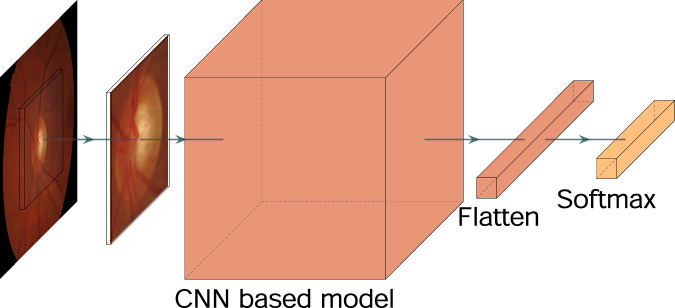
Basic block diagram of the proposed model to evaluate the PAPILA dataset.

Different CNN models were tested for feature extraction, then connecting the output to a fully connected layer and a *Softmax* classification layer, as depicted in Fig. 4. In particular, the following well-known pretrained models were selected: DenseNet121, VGG16, MobileNet, Inception, ResNet50 and Xcepcion. During the training stage, a class weighting in the loss function was performed according to the inverse of the class frequency, following the proportionality expression,

2 $${w}_{c}=\frac{N}{{N}_{c}\cdot C},$$

where *w*_*c*_ represents the weight for *c*-th class, *N* is the total number of samples, *N*_*c*_ is the number of samples of *c*-th class and *C* is the total number of classes.

Training was performed using a batch size of 16, with the Adam^50^ optimizer and a learning rate of 10^−4^. The cross entropy was used as cost function. The base CNN was pretrained with the *Imagenet*^51^ dataset. Additionally, to add diversity to the training set and improve the robustness in operation mode, a basic data augmentation was performed over the original training set: rescaling, horizontal flip, horizontal and vertical shift and zoom.

Each experiment has been evaluated using the Receiver Operating Characteristics (ROC) and the Area Under Curve (AUC) metric, which can be understood like illustrative measures to better observe the limits and possibilities of the dataset.

In addition, the *k-fold cross-validation* technique was used for assessing how the trained model generalizes to a separated dataset. A value of *k* = 5 was considered to be adequate for the size of the PAPILA dataset, and therefore the initial dataset was split into 5 folds, using one of them for testing and the remaining ones for training. Data from both eyes of a particular patient (fundus images or clinical information) were always included in the same fold. The model was then evaluated in these 5 scenarios computing all performance metrics, which are presented in this work in terms of mean value and standard deviation over the 5 folds.

#### Test #1. Multiclass eye classification

In this first experiment, each fundus image was considered as an independent unit, even when two images comes from the same patient. Note that according to Table 4, in some cases a patient could be diagnosed with early glaucoma in only one eye.

**Fig. 5:**
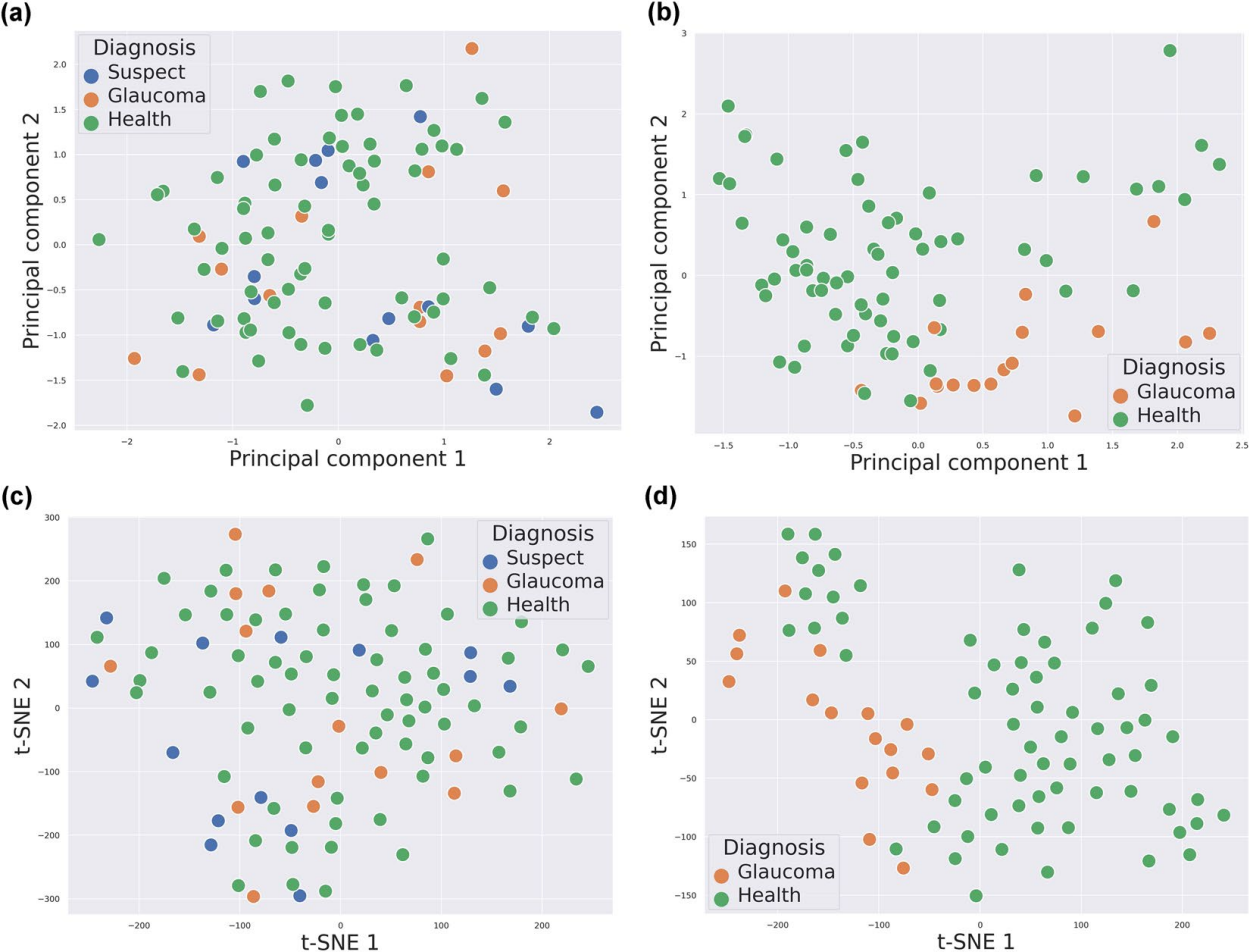
PCA and t-SNE projections of the post flatten layer (before entering to softmax) of ResNet-50 output vector for one fold, with the suspect class and without it. (**a**) PCA projections of three classes, (**b**) PCA projections of two classes, (**c**) t-SNE projections of three classes, (**d**) t-SNE projections of two classes.

After training, every retinal image in the test set was classified into the three classes that are present in the dataset: healthy, glaucoma and suspect. To illustrate this point, both a Principal Component Analysis (PCA) and a t-distributed Stochastic Neighbor Embedding (t-SNE) were performed for dimensionality reduction over the flattened output of a ResNet-50 as head in Fig. 4, when the model was trained using the *k-fold* cross-validation strategy and evaluated over an arbitrary fold. Figure 5 depicts the results of this projection over a 2D space, which shows (Fig. 5a,c) how the suspect class is not acting as an intermediate class between the healthy and glaucoma classes, hence it is noticeably mixed with both classes.

#### Test #2. Binary eye classification

According to the previous observation, the suspect class is not behaving as a borderline class between the healthy and the glaucoma classes, but rather as an unresolved class between them which makes it more difficult to find the separation boundary between classes. Then, for comparative purposes in this second experiment, the suspect class was removed from the original dataset and all the process was repeated under the same conditions of the previous case, except that now a *Softmax* classifier of only 2 units was used, and the vertical flipping was added to the data augmentation procedure to compensate for the lower number of samples in the dataset. The experimentation shows that, when this basic and common data augmentation is used, a remarkable improvement could be obtained for certain pretrained CNN heads (VGG16, MobileNet and Inception), while in others there is no positive improvement (DenseNet121, ResNet50 and Xception). This fact suggests that a specific data augmentation should be designed for each CNN architecture.

**Fig. 6:**
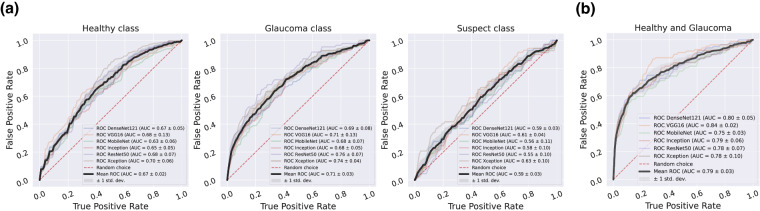
Classification baseline results of optical fundus images in PAPILA dataset. CNN reference models performance in terms of AUC metric computed from ROCs. (**a**) Test #1: Multiclass classification, (**b**) Test #2: Binary classification.

Figure 6 displays the ROC for both previous tests (Fig. 6a, multiclass classification, and Fig. 6b, binary classification). The AUC metric shows how the classification performance was clearly improved when the suspect class is not present. Furthermore, as shown in Fig. 5b,d, after eliminating the suspect class, we can see how the vast majority of the glaucoma samples of an arbitrary test set are clustered in the same area, showing a remarkable separability from the healthy samples. Both experiments were in any case affected by the problem of class imbalance, which represents a challenge for future research.

### Baseline results for clinical data and medical tests

The aim of this section is to illustrate the classification performance that can be achieved from the clinical data and medical tests included in the proposed dataset by means of diverse classical techniques. The results of this metadata classification can serve as a reference baseline for researchers in future investigations.

As in previous section, two scenarios are considered: multiclass eye classification and binary eye classification. In both cases, the following techniques have been chosen to evaluate the metadata dataset, because they represent methods of a different nature: (1) Logistic Regression, (2) k-Nearest Neighbors algorithm (k-NN), (3) Random Forest, and (4) Support-Vector Machine (SVM). Thus, logistic regression is a parametric model, K-NN is a nonparametric method, Random Forest is a method based on decision trees, and SVM is a semi-parametric method. In this way, the range of variability of these methods allows a more complete analysis, evaluation and discussion of results obtained with them.

#### Test #3. Multiclass eye classification

**Fig. 7:**
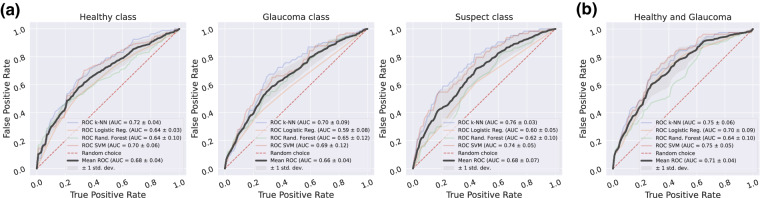
Classification baseline results of clinical data in PAPILA dataset. Standard models performance in terms of AUC metric computed from ROCs. (**a**) Test #3: Multiclass classification, (**b**) Test #4: Binary classification.

In this experiment, the instances of the three available classes in the PAPILA dataset are used to learn and to predict the class of every individual eye of the test subset, in a equivalent strategy to the case of fundus images. As can be observed in Fig. 7a, for a particular class (healthy, glaucoma or suspect) the four classification approaches perform quite similar. Note that the standard deviation of the AUC for the mean ROC measures the difference between the four methods, not between folds, and it is under 0.07 for the worst case. On the other hand, the mean AUC value between methods shows a poor and very uniform behaviour for the three class curves, ranging from 0.66 to 0.68.

#### Test #4. Binary eye classification

This test take into account only the samples of the healthy and glaucoma classes of the dataset to evaluate the AUC metric for eye classification. As can be observed in Fig. 7b, the AUC varies from 0.64 to 0.75, with a mean value between methods of 0.71, which constitutes a slight improvement compared to the previous multiclass scenario, although not so remarkable to that reached for the case of fundus images when glaucoma samples were removed from the dataset.

## Usage Notes

The PAPILA dataset contains medical data, retinal fundus images (with their corresponding segmentations of the optic disc and optic cup by two experts) along with their diagnosis. The main objective is to provide a comprehensive dataset to advance in the early diagnosis of glaucoma considering the joint information of both eyes of each patient. These resources are intended not only for healthcare professionals in the field of Ophthalmology, but also for researchers in the scientific community who develop computer tools to assist clinicians in, for example, diagnosing patients from fundus images or clinical data, optic disc and optic cup segmentation or even data augmentation using Generative Adversarial Networks (GAN). In the HelpCode folder, to facilitate future comparisons and ease the use of the dataset in basic machine learning tasks, instructions to assist other researchers with the reuse of the PAPILA dataset are given in a script and are exemplified in a Jupyter Notebook. The dataset splits used with the cross-validation technique are also indicated in that folder.

## Data Availability

The PAPILA dataset^44^ is publicly available at 10.6084/m9.figshare.14798004.v1. As detailed in the composition of the dataset, the clinical data of both eyes of each patient and the corresponding diagnosis are stored in spreadsheet and plain text format. In addition, the folder named HelpCode contains sample code in Python to read, handle and process the dataset. Jupyter Notebooks are also provided to exemplify the use of the PAPILA features.
