# Author Correction: PAPILA: Dataset with fundus images and clinical data of both eyes of the same patient for glaucoma assessment

**DOI:** 10.1038/s41597-024-03175-6

**Published:** 2024-04-17

**Authors:** Oleksandr Kovalyk, Juan Morales-Sánchez, Rafael Verdú-Monedero, Inmaculada Sellés-Navarro, Ana Palazón-Cabanes, José-Luis Sancho-Gómez

**Affiliations:** 1 Universidad Politécnica de Cartagena, 30202 Cartagena, Spain; 2 Hospital General Universitario Reina Sofía, 30003 Murcia, Spain

**Keywords:** Databases, Medical imaging, Optic nerve diseases, Databases, Medical imaging, Optic nerve diseases

Correction to: *Scientific Data* 10.1038/s41597-022-01388-1, published online 9 June 2022

In the Acknowledgements section, the funding source should have read ‘This work has been partially funded through the Spanish National projects AES2017-PI17/00771 and AES2017-PI17/00821 by Instituto de Salud Carlos III and by European Union (ERDF/ESF, “A way to make Europe”/ “Investing in your future”) and through regional project 20901/PI/18 by Fundación Séneca’. The original article has been corrected.

